# Challenging the ‘Culture’ around Blood Culture Ordering for the Adult Bone Marrow Transplant Inpatient Population

**DOI:** 10.1017/ash.2025.319

**Published:** 2025-09-24

**Authors:** Meg Edgerton, Ryne Wilson, Mark Juckett, Cate Gilbertson, Christina Dean, Cynthia Larson, Caprice Vanderkolk, Angela Kramer, David Pelletier, Jesse Sutherland, Christopher Linke, Alison Galdys

**Affiliations:** 1 M Health Fairview; 2 M Health Fairview - University of Minnesota Medical Center; 3 University of Minnesota Medical Center

## Abstract

**Background:** The adult bone marrow transplant (BMT) unit at an urban academic medical center in the Midwest reported the highest number of central line-associated bloodstream infection cases across the health system in 2022 and 2023 and notably had the second-highest volume of blood culture specimens collected when compared with other patient care units. Statistical analysis comparing BMT patients to a sample group of oncology patients with the same length of stay and central line days demonstrated that BMT patients had a median of 17 blood cultures per admission compared to 7 in the sample group (p-value 0.000). Moreover, a review of 21 weeks of BMT patient blood culture specimen results suggested that patients were undergoing cultures who were unlikely to have bacteremia or sepsis. **Method:** An interdisciplinary team created a nurse-driven, clinical decision-making algorithm to refine the release of blood cultures from a conditional order set for BMT patients. The objective of the algorithm was to safely reduce the number of blood culture specimens. It includes an updated fever threshold to align with national neutropenic fever guidelines, consideration for new-onset clinical instability, source of specimen collection, and time from the most recent blood culture. Analysis was completed on 827 cultures over 102 patient admissions in the pre-intervention period and 527 cultures over 162 patient admissions in the post-period. Balancing measures based on escalation of care were assessed by chart review. **Results:** When comparing blood culture specimens among BMT patients, the median specimen count per admission in the pre-intervention period was 6.0 (IQR = 3.5, 10.0), compared to 2.5 (IQR = 0.0, 5.0) specimens in the post-intervention period (p-value = 0.000). 37.7% of patient admissions were not cultured in the post-intervention period whereby 100% of patient admissions were cultured in the pre-intervention period. Of the 48 rapid responses, 10 intensive care unit transfers, and 1 code blue events in the 23-week post-intervention period, none were attributed to delayed detection of bacteremia or sepsis. **Conclusions:** Messaging that ordering providers should order fewer cultures is overly simplistic with consideration for the BMT patient population, yet diagnostic stewardship is essential to optimizing patient experience and outcomes. Attention to new clinical instability among BMT patients is important in detecting bacteremia. Stable symptoms in continuously observed BMT inpatients are unlikely to represent bacteremia.

